# Role of *Dgat2* in Glucose Uptake and Fatty Acid Metabolism in C2C12 Skeletal Myotubes

**DOI:** 10.4014/jmb.2307.07018

**Published:** 2023-08-18

**Authors:** So Young Bu

**Affiliations:** Department of Food and Nutrition, College of Engineering, Daegu University, Gyeongsan 38453, Republic of Korea

**Keywords:** Skeletal muscle, fatty acid, glucose, triacylglycerol, *Dgat2*

## Abstract

Acyl-coenzyme A (CoA):diacylglycerol acyltransferase 2 (DGAT2) catalyzes the last stage of triacylglycerol (TAG) synthesis, a process that forms ester bonds with diacylglycerols (DAG) and fatty acyl-CoA substrates. The enzymatic role of *Dgat2* has been studied in various biological species. Still, the full description of how *Dgat2* channels fatty acids in skeletal myocytes and the consequence thereof in glucose uptake have yet to be well established. Therefore, this study explored the mediating role of *Dgat2* in glucose uptake and fatty acid partitioning under short interfering ribonucleic acid (siRNA)-mediated *Dgat2* knockdown conditions. Cells transfected with *Dgat2* siRNA downregulated glucose transporter type 4 (*Glut4*) messenger RNA (mRNA) expression and decreased the cellular uptake of [1-^14^C]-labeled 2-deoxyglucose up to 24.3% (*p* < 0.05). Suppression of *Dgat2* deteriorated insulininduced Akt phosphorylation. *Dgat2* siRNA reduced [1-^14^C]-labeled oleic acid incorporation into TAG, but increased the level of [1-^14^C]-labeled free fatty acids at 3 h after initial fatty acid loading. In an experiment of chasing radioisotope-labeled fatty acids, *Dgat2* suppression augmented the level of cellular free fatty acids. It decreased the level of re-esterification of free fatty acids to TAG by 67.6% during the chase period, and the remaining pulses of phospholipids and cholesteryl esters were decreased by 34.5% and 61%, respectively. Incorporating labeled fatty acids into beta-oxidation products increased in *Dgat2* siRNA transfected cells without gene expression involving fatty acid oxidation. These results indicate that *Dgat2* has regulatory function in glucose uptake, possibly through the reaction of TAG with endogenously released or recycled fatty acids.

## Introduction

Fatty acids are bioactive chemicals that influence many physiological processes and are metabolized or stored as a variety of lipid species [1]. Triacylglycerols (TAG) are neutral lipids that serve as energy-storage molecules in most mammalian cells [1, 2]. In addition, TAGs act as metabolic sinks that trap free fatty acids and guard cells from the harmful effects of excessive free fatty acids and their subsequent metabolites [3, 4]. Indeed, defects in the metabolism of TAG in non-adipose tissues, including the liver and muscles, underlie the consequences of type 2 diabetes and fatty liver diseases [5-8]. For instance, free fatty acids from hepatic TAG impair insulin signaling and low-density lipoprotein (VLDL) secretion. In skeletal muscles and hepatocytes, defects in TAG synthesis lead to excessive free fatty acid-impaired glucose uptake [9] and cellular autophagy [10]. Hence, the factors involved in collecting fatty acids as storage lipids may be important for maintaining metabolic homeostasis and cellular functions. Even in skeletal muscles, fatty acids entering muscle tissues following uptake are partitioned into TAG before they are further metabolized or subjected to the mitochondrial oxidation [11].

Many factors are involved in channeling fatty acids toward and behind the TAG synthesis and breakdown [5, 12]. Acyl-coenzyme A (CoA):diacylglycerol acyltransferase (DGAT), a key TAG synthetic enzyme in the last complete stage, esterifies fatty acyl-CoAs to diacylglycerol (DAG). The synthetic role of DGAT and its high abundance and enzymatic activity in the adipose tissue, liver, and intestine are relatively well reported [7, 13, 14]. The two main DGAT isoforms in mammals, DGAT1 and DGAT2, mediate TAG synthesis by the same enzymatic process. These isoforms reside in the endoplasmic reticulum but belong to different protein families, and the consequences of their activity on lipid droplets are quite different, indicating dissimilar functions [15]. *Dgat1* and *Dgat2* share similar functions and compensate each other for TAG synthesis in adipocytes [16]. *Dgat1* knockout mice have a metabolically healthy profile despite a substantial decrease in TAG stored in adipose tissue and increased energy expenditure with enhanced glucose intolerance [17, 18]. In addition, TAG synthesis by DGAT1 protects the endoplasmic reticulum from lipotoxicity and inflammation in adipocytes via the lipolysis-re-esterification cycle of intracellular fatty acids [19]. Characterization of *Dgat2* does not seem reliable as *Dgat2* knockout in mice resulted in rapid death because of the impaired skin barrier and loss of the capacity to utilize glucose for thermogenesis in brown adipose tissue [20].

Furthermore, the role of DGAT in skeletal muscles is limited and has mostly been studied for *Dgat1* in cardiac muscles. Overexpression of *Dgat1* in the hearts of transgenic mice led to increase in the TAG level and reduction of ceramide, DAG, and free fatty acids by approximately 20–35% in cardiac muscles, without affecting heart muscle contraction [21]. In a cardiac myocyte-selective *Dgat1* transgenic mouse, reduced mitochondrial biogenesis and cardiac myopathy were observed with the downregulation of enzyme genes (cytochrome c oxidase 1 and cytochrome c) in mitochondrial oxidative phosphorylation [22]. Molecular inhibitors of *Dgat1* reduced the expression of genes that mediate fatty acid uptake and oxidation in human cardiac muscles and mouse myocytes [23, 24]. The upregulation of muscle-specific *Dgat1* increased triglyceride synthesis in skeletal muscles and attenuated diet fat-induced insulin resistance [25]. *Dgat1* knockout mice demonstrated insulin resistance because of a raised level of free fatty acids, whereas *Dgat1* overexpression suppressed protein kinase C, an inhibiting factor for insulin signaling, and c-Jun N-terminal kinase-1, consequently increasing glucose transporter 4 (*Glut4*) level in the skeletal muscle [26].

Compared with *Dgat1*, which has been relatively well characterized in many studies, the phenotype of *Dgat2* deficiency seems to vary depending on the tissue, mammalian species, and experimental conditions. A previous study reported that overexpressed murine *Dgat2* was found in mitochondria-associated membranes [27], suggesting a possible role for this gene in substrate oxidation. However, the *Dgat2* localization study was performed in CV-1 (simian) in Origin and carrying the SV40 genetic material (COS) cells (a cell line typically used in transfection studies of vectors/plasmid-containing genes of interest), and could not accurately reflect physiologically relevant conditions. Recently, one study reported that suppressing this enzyme specifically has been tested in human skeletal muscle using small inhibiting chemicals targeting DGAT1 and DGAT2 [28]. The results showed that DGAT1 predominantly utilizes exogenously introduced fatty acids for TAG synthesis, whereas DGAT2 seems to be more involved in utilizing fatty acids from de novo synthesis. A study with transgenic mice overexpressing human DGAT2 in glycolytic skeletal muscle, which stores less TAG than oxidative muscle [29], showed excessive lipid accumulation with impaired insulin signaling. However, the *Dgat2*-mediated fate of fatty acid metabolism and the consequences of glucose uptake in skeletal muscle have yet to be investigated. Owing to the importance of skeletal muscle as a major tissue for clearing more than 80% of the prandial glucose [30] and 50–60% of the free fatty acids [31], the role of *Dgat2* in skeletal muscle should be further investigated. Therefore, this study aimed to explore the role of *Dgat2* in glucose uptake and fatty acid metabolism using a short interfering ribonucleic acid RNA (siRNA)-mediated knockdown of *Dgat2* in C2C12 mouse-derived skeletal muscle cell lines.

## Materials and Methods

### Materials

Cell culture equipment, including plates and pipettes, were bought from Corning Inc. (USA), or SPL Life Sciences (Korea). The tissue culture medium was bought from Thermo Fisher Scientific (USA). Lipid standards (TAG, DAG, phosphatidyl choline) for thin-layer chromatography (TLC) were purchased from Sigma-Aldrich (USA). Lipid sampling tubes were obtained from SamwooKurex (Korea). Additionally, [1-^14^C]-labeled fatty acids or acetic acids were purchased from PerkinElmer Life Sciences (USA). Unless otherwise indicated, other chemicals were obtained from Sigma-Aldrich or Junsei Chemicals (Japan).

### Cell Culture

The C2C12 mouse myoblast cell line was purchased from the American Type Culture Collection (ATCC)(USA). Cells were maintained in Dulbecco’s modified Eagle’s medium (DMEM) supplemented with 10% fetal bovine serum (FBS), 50 IU/ml penicillin, 50 μg/ml streptomycin, and 5.5 mM glucose in a water-jacketed, 37°C incubator supplied with 5% CO_2_. The “Day 0” set when the cells reached ~100% confluence. Then, the 2% horse serum was added to the cell culture medium to differentiate into skeletal muscle myotubes.

### RNA Interference

The siRNA targeting *Dgat2* isoforms was purchased from Qiagen (USA). The two siRNA sequences were selected to target mouse *Dgat2* (NM_026384). Targeted nucleotide positions were 660–680 and 498–528 for *Dgat2*-a and *Dgat2*-b, respectively. AllStars siRNA, which targets non-specific sequences, was designed by Qiagen (USA) and used to set up control cells. C2C12 cells are spontaneously dividing pre-myoblast cells. Hence, transfection with the siRNA was accomplished after the cells had fully differentiated and stabilized as skeletal muscle fibers. Briefly, the differentiated C2C12 muscle fibers were transfected with siRNA at 250 ng siRNA/cell, which covered a 3.5 cm^2^ area of culture plate, using commercially available transfection reagent (HiPerfect, Qiagen, USA) after the cells were fully confluent. At 6 h after siRNA transfection, media were switched with DMEM containing 2% of horse serum unless otherwise noted. During the experiment, over 90% of cell viability was confirmed for both control and *Dgat2* siRNA-transfected cells.

### Cell Viability Assessment

Changes in cell viability following siRNA transfection were measured using a 3-(4,5-dimethylthiazol-2-yl)-2,5-diphenyltetrazolium bromide (MTT) assay kit, as previously reported [9]. Briefly, the siRNA-transfected cells were reacted with MTT reagent for 1 h, and the produced colored formazan products were analyzed using a spectrophotometer (450 nm).

### Real-Time Polymerase Chain Reaction (PCR)

The cells were collected at the designated time points during the experiment, and total cellular RNA was isolated with TRIzol reagent (Thermo Fisher Scientific) following the product manual. Isolated RNA was stored at below than -80°C until investigation. The RNA was quantified and the quality was confirmed by the values of the optical density obtained at 260 nm and 280 nm using a SimpliNano spectrophotometer (General Electric [GE], USA). Synthesis of copy deoxyribonucleic acid (cDNA) was initiated using the SuperScript III reverse transcriptase and random hexamer primers (Clontech, USA). The synthesized cDNAs were mixed with 2X Power SYBR Green PCR Master Mix (Thermo Fisher Scientific) and subjected to real-time PCR quantification (StepOnePlus, Thermo Fisher Scientific). Gene-specific primers used for PCR analysis are listed in Table 1. Expression of each gene was quantified using the delta-delta cycle threshold (Ct) method, and the quantified results were reported relative to ribosomal protein L32 (*Rpl-32*).

### Glucose Uptake and Clearance Measurement

Differentiated C2C12 muscle fibers transfected with either *Dgat2*-a or *Dgat2*-b siRNA were fasted in a serum-depleted medium for 4 h. The cells were washed with phosphate-buffered saline (PBS) and loaded with a glucose mixture composed of 50 μM 2-deoxyglucose and 1.0 μCi 2-deoxy-D-[1-^14^C]-glucose for 1 h. The reaction media contained 10 nM insulin with 10 nM dexamethasone. After washing, the cells were lysed in PBS containing 1%Triton X-100. Radioactivity of the released ^14^C-labeled 2-deoxyglucose in cell lysates was detected using a Beckman Coulter scintillation counter (LS6500IC, USA). To investigate glucose clearance in the media, cells were treated with high glucose (25 mM) in the presence of 10 nM insulin at 48 h after transfection, and the media were harvested at the specified time points (0, 15, 30, and 60 min after glucose treatment). The glucose concentration in the cell culture media relative to the media of control cells was quantified using a colorimetric method (Fujifilm Wako Chemicals, USA).

### Radioisotope Labeling Studies for Measuring Fatty Acid Metabolism

For pulse-chase experiments, differentiated C2C12 cells were treated with 1.0 μCi of [1-^14^C]-oleic acid-bovine serum albumin (BSA) with 500 μM oleic acid-BSA in 1 ml DMEM for 2 h and harvested (pulse). After 2 h of pulse, cells were rinsed twice with 1% BSA and maintained for 6 h in fatty acid-free media containing only 1 mM carnitine (chase). The fatty acid chasing cells were then collected and subjected to total lipids extraction [32]. Extracted lipids were separated by TLC using silica gel G plates (0.25-mm, Sigma, USA). A solution of hexane : ethyl ether : acetic acid = 80 : 20 : 1 (v/v), was used as a separate solvent for cellular lipids and commercial lipid standards. The separated lipids were marked with iodine fog and gels containing separated lipids were scraped. Radiolabeled fatty acids in the scraped parts were measured using a Beckman Coulter liquid scintillation counter.

### Measurement of Fatty Acid Oxidation

Fatty acid incorporated into β-oxidation products, acid-soluble metabolites (ASM), and CO_2_ levels were measured as described previously [9]. After cells were transfected, they were treated with BSA-bound 500 μM [1-^14^C]-oleic acids in sealed plates at 37°C. For the measurement of the ASM, the medium was acidified with 70% perchloric acid and centrifuged at maximum speed (>10,000 ×*g*) for at least 10 min to obtain the particle-free aliquots. Next, the radioactivity of ^14^C-labeled ASM in 200 μl of the supernatant was quantified with a scintillation counter. For CO_2_ measurement, the filter paper (Whatman No.1) was saturated with 1 M sodium hydroxide for 1 h and CO_2_ from 200 to 400 μl of medium was trapped on that filter paper and added with a half volume of 70% perchloric acid. The amount of ^14^C-labeled CO_2_ on the trapped filter paper was determined using scintillation counting.

### Western Blotting

Cells were harvested in lysis solution (pH 7.4) composed of 0.1% Triton X-100, 10 mM Tris-HCl, and 150 mM NaCl with a phosphatase/protease inhibitor cocktail. Aliquots of lysed proteins (10–25 μg) were denatured by adding and boiling with sample buffer (pH 6.8) containing 15% β-mercaptoethanol, 50 mM Tris, 2% sodium dodecyl sulfate (SDS), 10% glycerol, and 1% bromophenol blue. The denatured proteins were separated by 8%SDS-Poly Acrylamide Gel electrophoresis (PAGE) and electro-transferred to a polyvinylidene difluoride (PVDF) membrane (Merck Millipore, USA). Equal transfer of proteins was confirmed using Ponceau S staining. The blotted membrane was blocked with 1% fish-gelatin in Tris-buffered saline (TBS) (pH 7.4) with 0.05% Tween 20 and then incubated with p-Akt, Akt (Cell Signaling, USA), or β-actin (Sigma, USA). Proteins of interest were identified using enhanced chemiluminescence (ECL) reagents after incubation with a secondary antibody coupled with horseradish peroxidase (Santa Cruz Biotechnology, USA). Images were developed using a LAS 4000 image analyzer (GE Healthcare, USA). The density of protein was enumerated using the ImageJ software (National Institutes of Health [NIH], USA).

### Statistical Analysis

Over the course of the study, experiments were performed separately at least two times on cultured plates. Data obtained from all repeated experiments are reported as the means ± standard error of the mean (SEM). Statistical analyses were performed using Statistical Analysis Software (SAS) 9.4 (SAS Institute Inc. USA). The significance of the data comparison was determined by Student’s *t*-test and declared at *p* < 0.05.

## Results

### Baseline Confirmation for Skeletal Myocyte Differentiation and *Dgat2* Expression

**Fig. 1:**
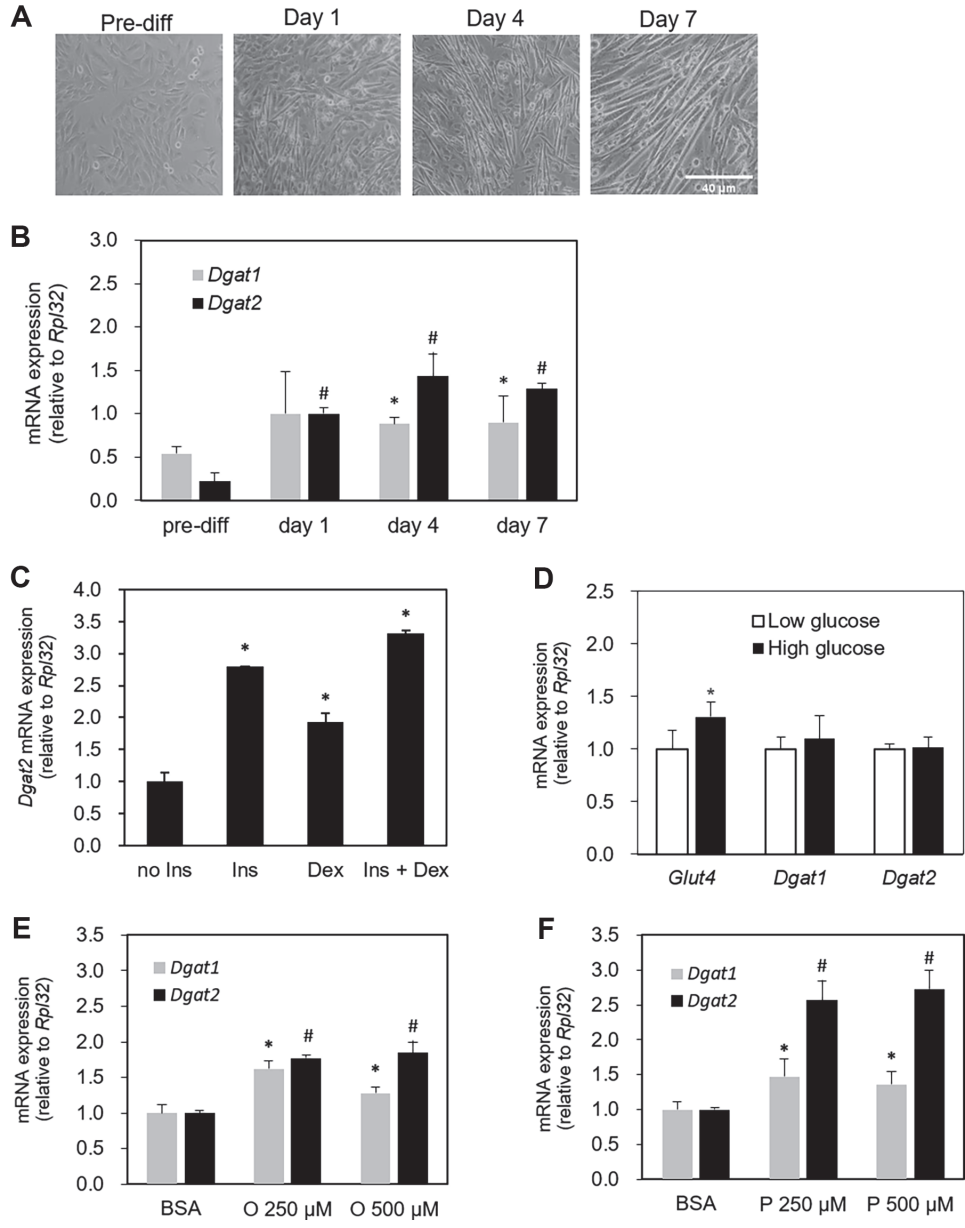
Baseline confirmation for skeletal myocyte differentiation and *Dgat2* expression. Mouse-derived C2C12 cells were cultured and harvested before and at one, four, and seven days after reaching 90–100% confluence, and (**A**) the representative images of cell morphology (20x) during the differentiation, (**B**) the mRNA expression of *Dgat2* were obtained. The abundance of *Dgat2* in response to (**C**) insulin or dexamethasone, (**D**) high glucose (25 mM glucose), and (**E, F**) high fatty acids (oleic acid or palmitic acid) was measured in differentiated skeletal myotubes four days after confluence. Gene expression values on all investigated days were normalized to the expression value of (**B**) day one, (**C**) no insulin control, (**D**) basal glucose (5 mM), or (**E, F**) BSA treatment. The data are expressed as the means ± SEM. Superscripts * and # indicate *p* < 0.05, vs. day one (graph **B**), no insulin (graph **C**) or BSA-only treatment (graph **E, F**) for *Dgat1* and *Dgat2*, respectively. * *p* < 0.05 vs. basal glucose (graph **D**). Ins, insulin; Dex, dexamethasone; BSA, bovine serum albumin; O, oleic acid; P, palmitic acid.

Initially, we confirmed that the C2C12 cells were fully differentiated over the course of the experiment (Fig. 1A) and the morphology of differentiated C2C12 skeletal myotubes was similar or identical to previously reported images of the well-differentiated C2C12 cells [33, 34]. In the present study, the expression levels of *Dgat2* messenger RNA (mRNA) gradually increased during C2C12 cell differentiation (Fig. 1B). In pre-myogenic cells, *Dgat2* mRNA was expressed at a low level and highly increased upon the launch of differentiation through the course of cellular differentiation, which fully corresponded to the experimental timeframe of this study (Fig. 1B). *Dgat1* expression tended to increase during cellular differentiation; however, the extent of increase at day 1 was not statistically significant compared to the pre-differentiated period (Fig 1B). The *Dgat2* expression was strongly induced by both insulin treatment as well as dexamethasone (Fig. 1C). Furthermore, we checked to see if the cell viability or cellular morphology had changed by visible observation during the time course (6 h) of high glucose or fatty acid treatment in each experiment. Previously, cytotoxicity in C2C12 cells had not been reported in the experiment [35, 36]. Upon high glucose treatment, *Glut4* mRNA expression increased. Still, no induction of *Dgat2* or *Dgat1* was observed (Fig. 1D). The level of *Dgat2* mRNA was upregulated by both oleic acid and palmitic acid treatment. Its induction was more pronounced in cells treated with palmitic acid, which showed up to two-to-three-fold induction relative to BSA-treated cells (Fig. 1E and 1F). The level of *Dgat1* mRNA expression in response to either of the fatty acids was similar to the level of *Dgat2* mRNA expression, but the fold-induction of *Dgat1* expression was similar between the oleic and palmitic acid treatments.

### Confirmation of *Dgat2* Suppression by *Dgat2* siRNA

**Fig. 2:**
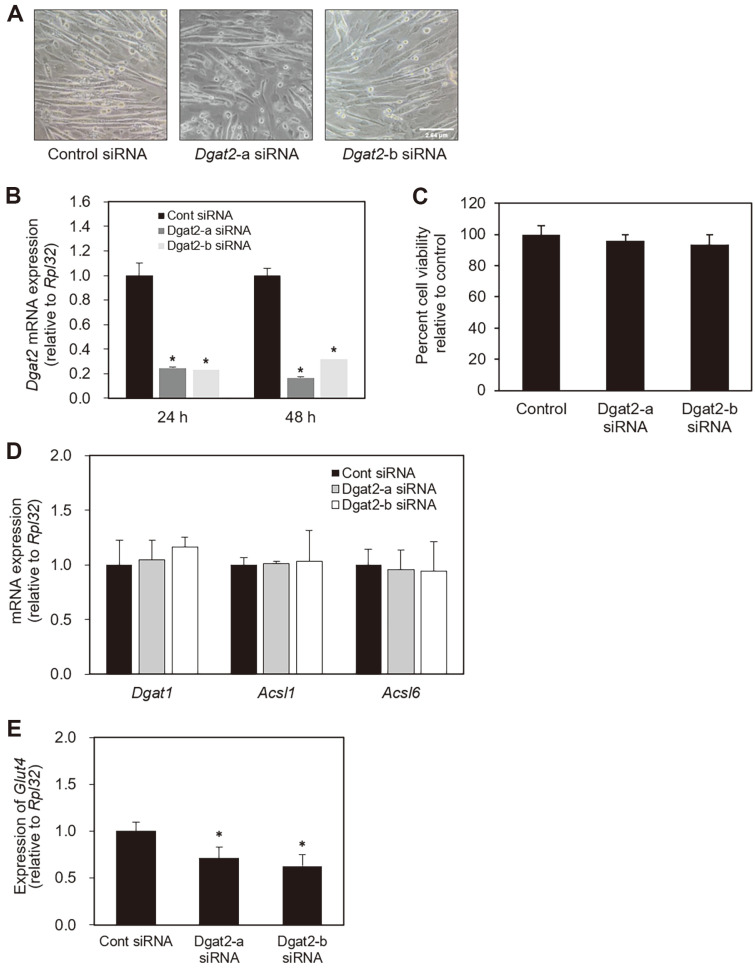
Confirmation of *Dgat2* suppression by *Dgat2* siRNA. (**A**) Differentiated C2C12 cells were transfected with control, *Dgat2*-a, or *Dgat2*-b siRNAs. Total RNA was extracted 24 and 48 h after transfection. (**B**) The abundance of *Dgat2* mRNA was quantified by quantitative real-time (RT)-PCR and normalized to *Rpl-32*. Data are expressed as arbitrary values for control siRNA-transfected cells. (**C**) Cell viability assessed 72 h after siRNA transfection is presented. (**D**) The mRNA abundances of the *Dgat1*, *Acsl*1 and *Acsl6* were measured 52 h after transfection. (**E**) The mRNA abundances of the *Glut4* were measured 52 h after transfection. The data are expressed as the means ± SEM. * *p* < 0.05, compared with controls.

We validated the knockdown efficiency of the two optimal siRNA sequences targeting *Dgat2*. Transfection of either control or *Dgat2* siRNA was performed after confirmation of C2C12 cells being differentiated (Fig. 2A). The level of *Dgat2* mRNA was substantially reduced by more than 70% in the cells transfected with *Dgat2*-a or *Dgat2*-b siRNA (Fig. 2B). In addition, we confirmed that over 90% of cells were viable at 72 h after *Dgat2* siRNA transfection (Fig. 2C). Then, we examined whether fatty acid transporter or glucose transporter proteins expression was altered due to *Dgat2* knockdown by analyzing the mRNA expression for Acyl-CoA synthethase (*Acsl*)1 and *Acsl6*, previously reported acyl-CoA synthethase isoforms investigated in skeletal muscle [9, 37]. The *Acsl*1 and *Acsl6* mRNAs were not significantly affected by either siRNA sequence targeting *Dgat2* (Fig. 2D). In contrast to no changes in the expression of genes involving fatty acid channeling, *Glut4* mRNA showed ~40% less induction upon insulin treatment in *Dgat2* siRNA-transfected cells compared to control cells. The level of *Dgat1* mRNA was not changed in *Dgat2* siRNA transfected cells, indicating no compensational expression of *Dgat1* in C2C12 cells upon siRNA-mediated knockdown of *Dgat2*.

### Glucose Uptake in C2C12 Cells Following *Dgat2* Knockdown

**Fig. 3:**
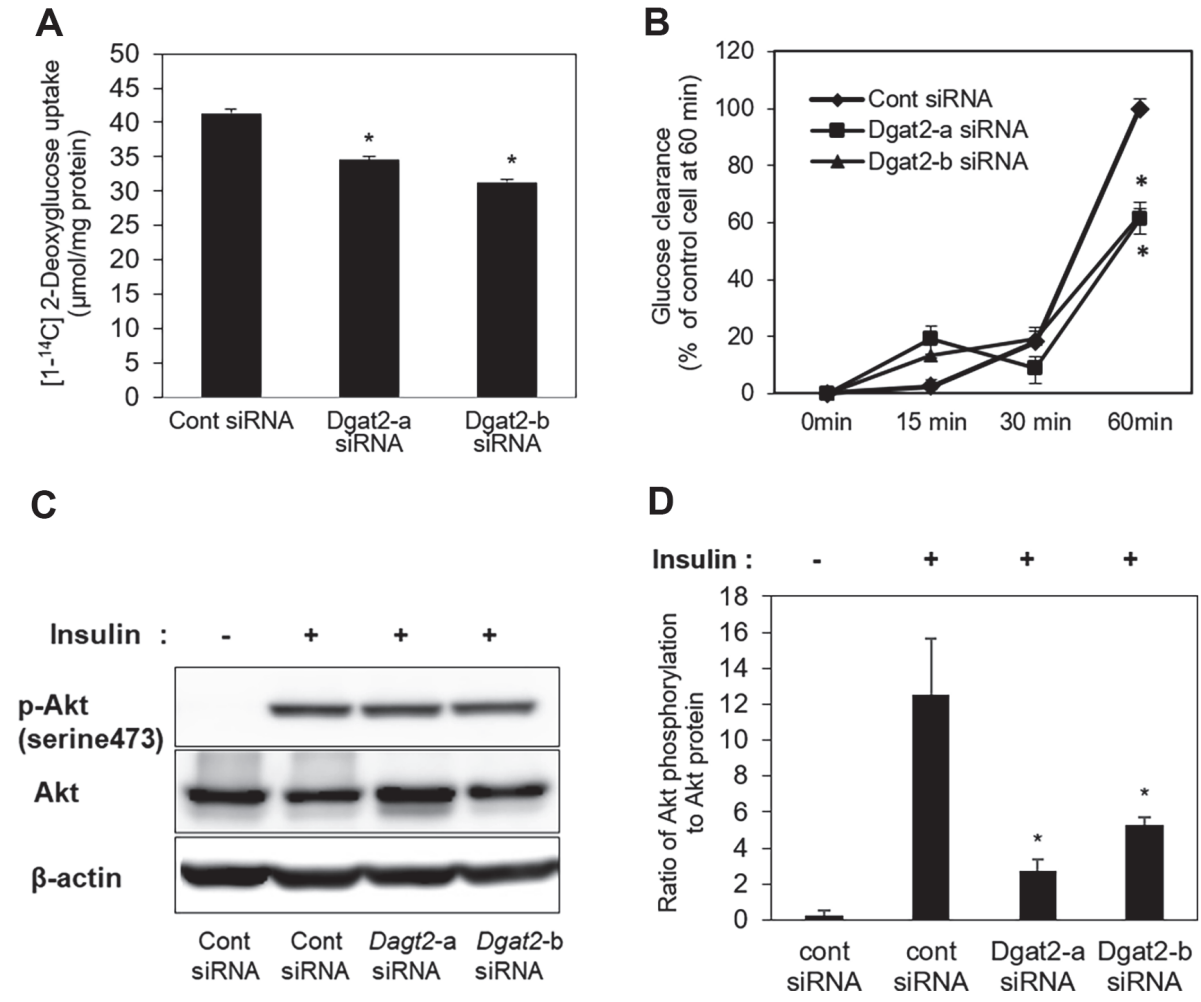
Glucose uptake in skeletal myotubes under *Dgat2* knockdown. Three days after the initiation of differentiation, cells were transfected with control, *Dgat2*-a, or *Dgat2*-b siRNA. At 52 h after the transfection of the *Dgat2* siRNA, the cells were fasted for 4 h, and (**A**) the cells were labeled with [1-^14^C]-2-deoxyglucose for 1 h in the presence of insulin (10 nM). Then, the [1-^14^C]-2-deoxyglucose uptake was measured. To investigate glucose in the remaining media, cells were treated with 10 nM insulin and 10 nM dexamethasone 52 h after transfection. (**B**) The medium was harvested at the indicated time points (0, 15, 30, and 60 min), and the remaining glucose was quantified. At 52 h after the transfection of either *Dgat2*-a or *Dgat2*-b siRNAs, (**C**) phosphorylation of Akt in cells transfected with control or *Dgat2* siRNA was measured and (**D**) the induction of Akt phosphorylation in response to insulin was calculated by the relative density of the protein band to the nonphosphorylated form. The data are expressed as the means ± SEM. * *p* < 0.05, compared with control siRNAs.

To explore whether decreased *Glut4* expression affects glucose uptake under *Dgat2* knockdown, siRNA-transfected cells were incubated in serum-depleted media for 4 h, followed by [1-^14^C]-2-deoxyglucose uptake assays. The cellular uptake of radioisotope labeled 2-deoxyglucose was significantly decreased by *Dgat2* siRNA upon insulin treatment (Fig. 3A). We further observed glucose clearance from the media at 0, 10, and 30 min after the insulin treatment. In comparison to control cells, *Dgat2* knockdown cells showed a significantly lower degree of glucose clearance in a time-dependent manner (Fig. 3B). These changes coincided with decreased Akt phosphorylation following *Dgat2* knockdown (Fig. 3C and 3D).

### Initial Fatty Acid Partitioning Under *Dgat2* Suppression

**Fig. 4:**
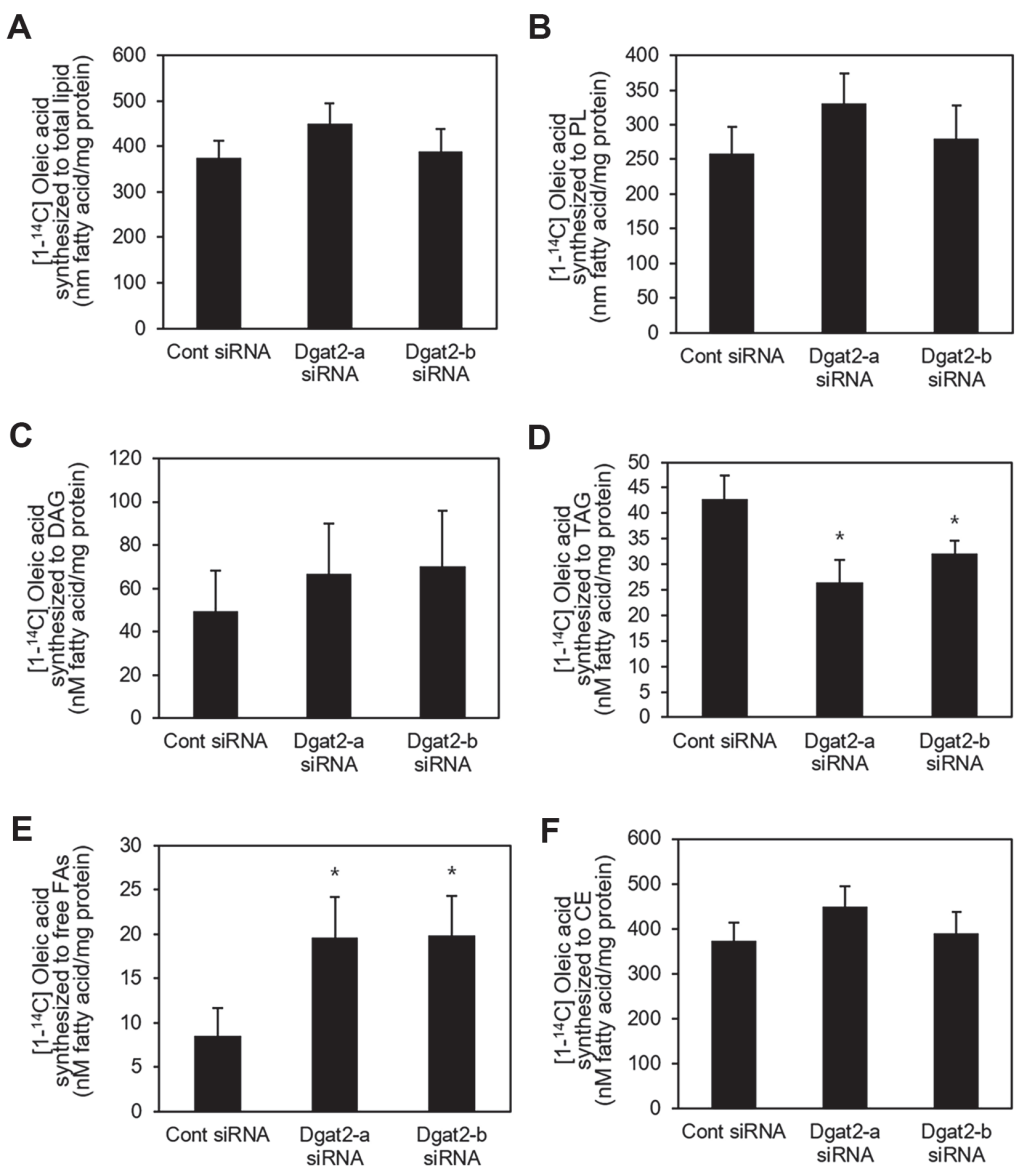
Initial fatty acid partitioning under *Dgat2* suppression. Differentiated C2C12 skeletal myotubes were transfected with *Dgat2* siRNA (*Dgat2*-a or *Dgat2*-b). Cells were treated with 500 μM oleic acids labeled with 1.0 μCi [1-^14^C]- oleic acids per 0.5 × 10^6^ cells for 2 h to trace the intracellular fatty acids. Lipids were extracted from cells. (**A**) [1-^14^C]-oleic acid incorporation into the total lipid content, and (**B**) PL, (**C**) DAG, (**D**) TAG, (**E**) free fatty acids, and (**F**) cholesteryl esters were quantified. Data are expressed as the means ± SEM from two experiments performed in triplicate. * *p* < 0.05, compared with control siRNAs.

Based on the reported function of Dgat isoforms in accumulation of lipids including TAG in adipocytes and hepatocytes [15, 16] and the finding of this study that *Dgat2* suppression decreased glucose uptake, the effect of *Dgat2* deficiency on fatty acid metabolism by tracking [1-^14^C]-oleic acid in differentiated C2C12 skeletal muscle fibers was investigated. C14-labled oleic acid integration into the total intracellular lipids was not significantly different among cells transfected with either control, *Dgat2*-a, or *Dgat2*-b siRNA after 2 h incubation with oleic acids. The incorporation of C14-labeled oleic acid did not differ between cells transfected with either *Dgat2* or control siRNA. Both *Dgat2*-a and *Dgat2*-b siRNAs significantly decreased the C14-labled oleic acid integration into TAG, by up to 27.9% and 24.9%, respectively, in comparison to the control cells (Fig. 4B). However, the levels of [1-^14^C]-oleic acid synthesized into phospholipids (PL) and DAG in *Dgat2*-deficient cells were similar to those in control cells (Fig. 4C and 4D). The amount of ^14^C-labeled free fatty acids in *Dgat2*-deficient cells was significantly higher than that in control siRNA-transfected cells (Fig. 4E). The amount of [1-^14^C]-oleic acid synthesized into cholesteryl esters was not significantly altered in *Dgat2* siRNA-transfected cells (Fig. 4F).

### Alteration of Intracellular Fatty Acid Turnover Under *Dgat2* Suppression

**Fig. 5:**
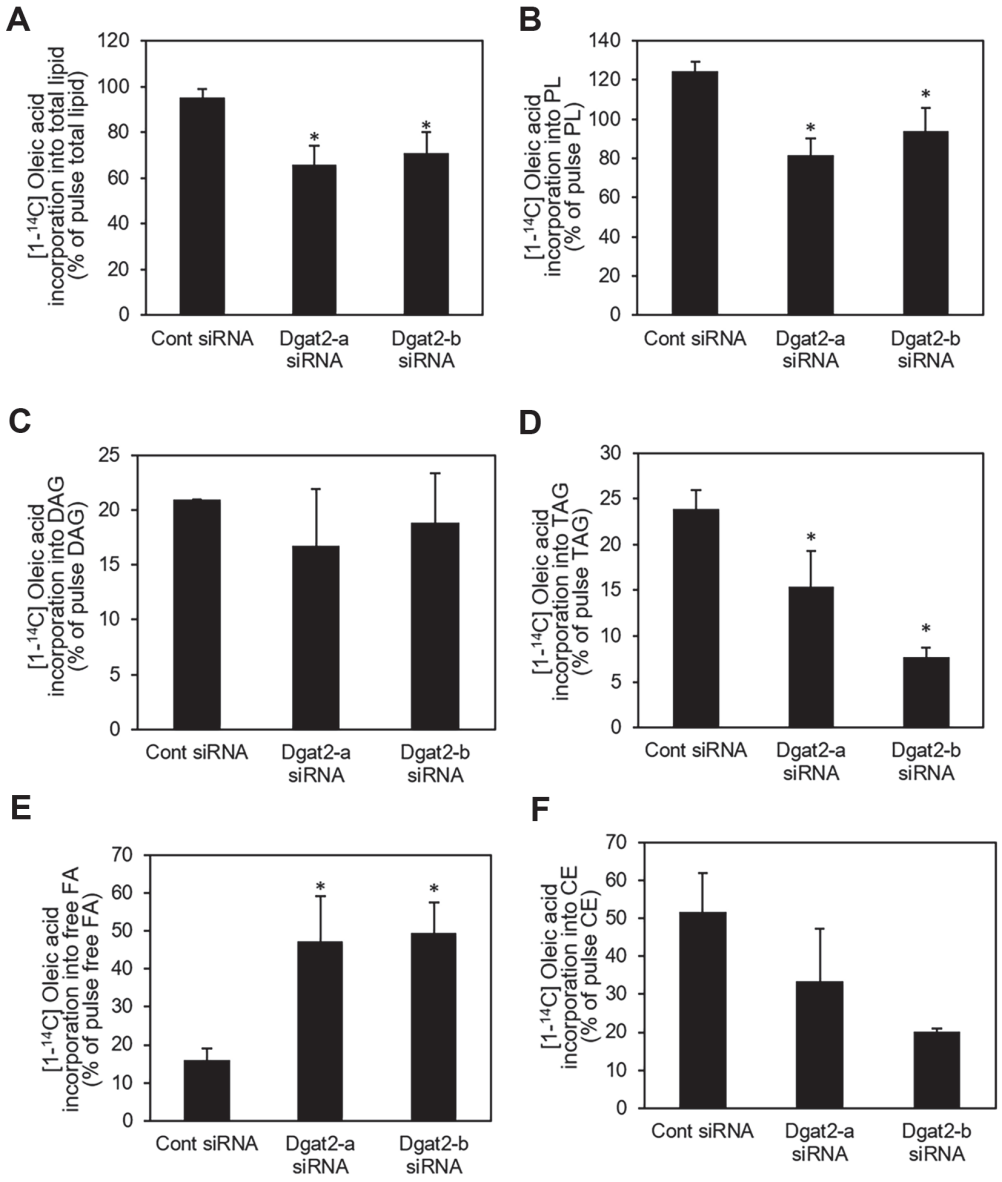
Effects of *Dgat2* knockdown on intracellular fatty acid turnover. Differentiated skeletal myotubes transfected with control, or *Dgat2* siRNA duplexes, were treated with 500 μM oleic acids and 1.0 μCi [1-^14^C]-oleic acids. After 2 h of labeling with 500 μM oleic acids and 1.0 μCi [1-^14^C]-oleic acid, the cells were washed and incubated in a medium containing no fatty acids for 6 h (chase). The [1-^14^C]-oleic acids remaining in (**A**) total lipids, (**B**) TAG, (**C**) PL, (**D**) DAG, (**E**) intracellular free fatty acids, and (**F**) cholesteryl esters were quantified. (**A–F**) The data are presented as percentage (%) of total pulse lipids that were quantified, at which time the cells were harvested after a 2 h pulse of C14-labeled oleic acids. All data are expressed as the mean (SEM) from two experiments performed in triplicate. *p < 0.05, compared with control siRNAs.

Intracellular lipids undergo dynamic alteration via hydrolysis and remodeling [11, 38]. Thus, we determined whether *Dgat2* altered lipid turnover by chasing the cellular fatty acids after a pulse loading of ^14^C-labeled fatty acids. The release of ^14^C-labeled fatty acids from total lipids, TAG, and PL increased by either *Dgat2*-a or *Dgat2*-b siRNA (Fig. 5A-5C). The amount of fatty acid loss from DAG did not considerably change by either of *Dgat2* siRNAs (Fig. 5D). The suppression of *Dgat2* decreased the level of ^14^C-labeled lipids in total lipids, TAG, and PL in cells up to 31.0, 67.6, and 34.4%, respectively, compared to control cells (Fig. 5A-5C). In contrast to storage lipids, *Dgat2* siRNA increased the amount of intracellular free fatty acids by three-fold relative to control cells after a pulse of radiolabeled- and cold oleic acids (Fig. 5E). *Dgat2* siRNA decreased ^14^C-labeled intracellular cholesteryl esters by up to two-fold. Still, the change was not statistically significant (Fig. 5F). Decreased ^14^C-labeled total lipids, TAG, and PL coincided with the increase of ^14^C-labeled free fatty acids during lipid turnover indicating that cells could not re-esterify cellular free fatty acids toward TAGs or PLs during *Dgat2* suppression. Transient knockdown of *Dgat2* leads to reduced TAG levels, indicating its limited capacity for the reacylation of fatty acids into TAG.

### Changes in Fatty Acid Oxidation by *Dgat2* Knockdown

**Fig. 6:**
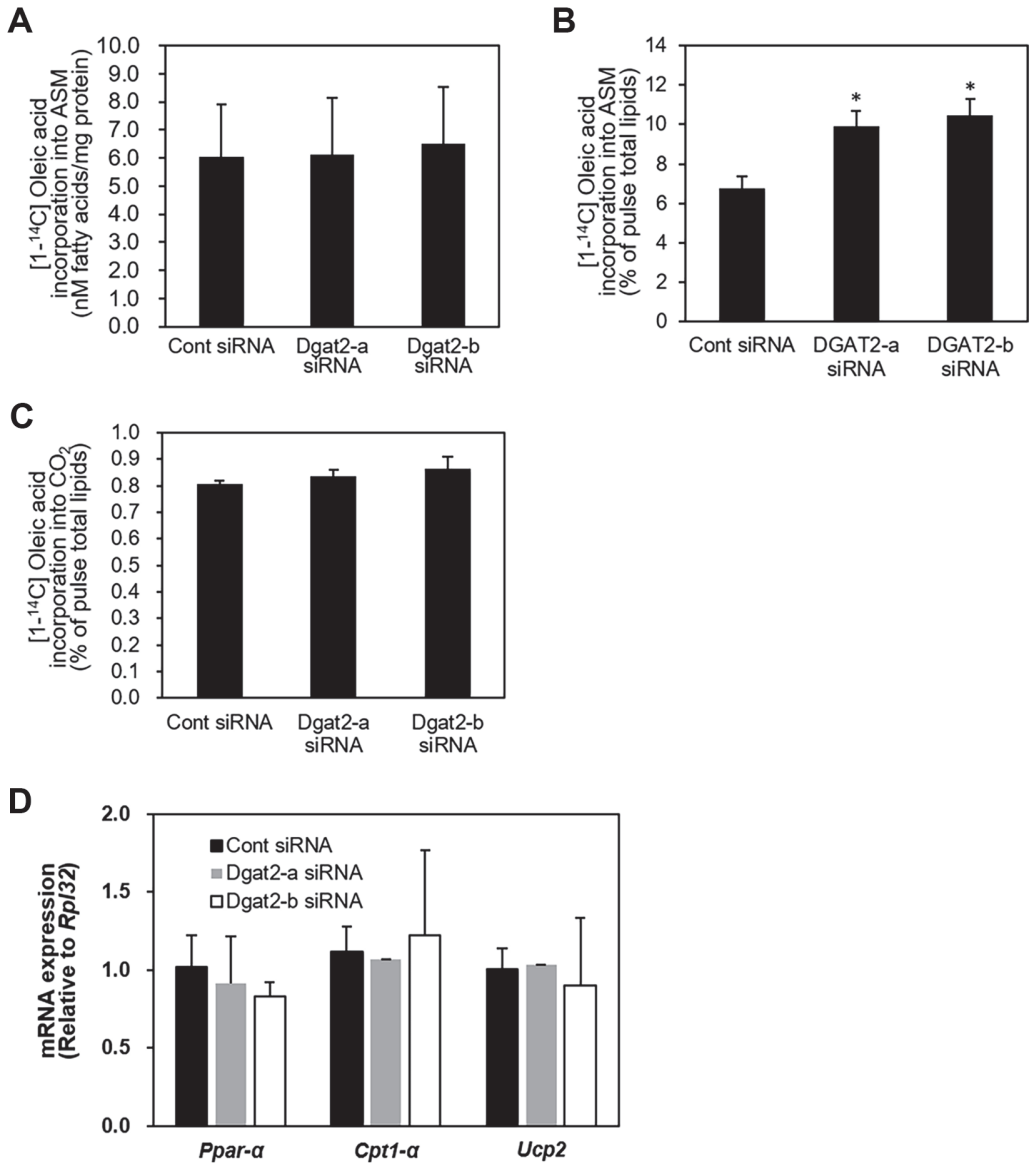
Changes in fatty acid oxidation by *Dgat2* knockdown. Differentiated skeletal myotubes transfected with control, or *Dgat2* siRNA duplexes, were treated with 500 μM oleic acids and 1.0 μCi [1-^14^C]-oleic acids for 2 h. The medium was collected, and oxidation of [1-^14^C]-oleic acids to a) acid-soluble metabolites (ASM) was quantified. After 2 h of labeling with 500 μM oleic acids and 1.0 μCi [1-^14^C]-oleic acid, the cells were washed and incubated in a medium containing no fatty acids for 6 h (chase). The oxidation of [1-^14^C]-oleic acids to (**A, B**) ASM and (**C**) CO_2_ was quantified. (**D**) The expression levels of the peroxisome proliferator-activated receptor alpha (*Ppar-α*), carnitine palmitoyltransferase 1 alpha (*Cpt1-α*), and uncoupling protein 2 (*Ucp2*) mRNA levels were measured. The data are presented as percentage (%) of pulsed total lipids, quantified when the cells were harvested after a 2 h pulse of ^14^C-labeled oleic acids. All data are expressed as the mean (SEM) from two experiments performed in triplicate. *p < 0.05, compared with control siRNAs.

Because knockdown of *Dgat2* resulted in accumulation of intracellular fatty acids, we assessed the degree of fatty acid utilization for mitochondrial oxidation during chase by measuring ^14^C-radioistope-incorporated acid-soluble metabolites. During the pulse period, *Dgat2* suppression did not change the oxidation of [1-^14^C]-oleic acid to ASM (Fig. 6A). The deficiency of *Dgat2* increased the integration of [1-^14^C]-oleic acids to ASM by 6-10%relative to control condition. Suppression of *Dgat2* did not alter the radiolabeled-oleic acid conversion to the CO_2_ (Fig. 6B and 6C). The mRNA expression levels of *Ppar-α*, *Cpt1-α* and *Ucp2*, genes involved in the mitochondrial oxidation process, did not change (Fig. 6D).

## Discussion

In this study, we investigated the consequence of transient *Dgat2* knockdown in glucose uptake and fatty acid channeling in C2C12 cells. The siRNA-induced knockdown of *Dgat2* in C2C12 skeletal myotubes decreased both glucose uptake and the degree of fatty acid partitioning into total lipids and TAG. The results of this study confirm the previous findings regarding an association between fatty acid metabolism and glucose uptake in skeletal muscle and suggest an intervening role of *Dgat2* in fatty acid metabolism of differentiated C2C12 skeletal myotubes.

At the beginning of this study, the differentiation of C2C12 cells was confirmed. Our results showed that the *Dgat2* expression was increased during the course of C2C12 cell differentiation. The findings of our study are consistent with our previous finding that *Acsl6*, a fatty acyl-CoA-metabolizing gene, was highly expressed in C2C12 cells during differentiation [9]. The transformation of myoblasts into differentiated skeletal myotubes coincides with the development of several transporters and genes involved in energy metabolism [39] as well as with myogenic regulatory factors, myosin heavy chains, and fiber proteins [33]. A recent study showed that lipid droplet formation increased during C2C12 cell differentiation and in turn, contributed to the differentiation process of C2C12 cells [34]. Because *Dgat1* and *Dgat2* are essential genes for TAG synthesis based on the enzymatic role of their protein, the increased *Dgat1* and *Dgat2* expression in differentiated C2C12 cells indicates that, as lipid droplets did to differentiation process, Dgat genes, along with upregulation of fatty acid transport or activating genes, may contribute to the contraction or energy-producing function of differentiated skeletal muscle fiber by regulating energy substrate usage and storage.

Although expression patterns of *Dgat2* and *Dgat1* during cellular differentiation were not much different in this study, the level of *Dgat2* mRNA expression in response to high fatty acids differed from that of *Dgat1*. The mRNA level of these genes increased in response to either oleic or palmitic acids. The *Dgat2* expression increase in response to palmitic acids was more pronounced than that in cells treated with oleic acid, while the induction level of *Dgat1* expression was similar between these cells. These results indicate that *Dgat2* may have more preference to saturated fat because the palmitic acids are a usual primary type of fatty acids derived from de novo lipogenesis [40]. The dose of fatty acids we used in this study was relatively high compared to that in previous studies using C2C12 cells [35, 36]. Our ongoing study, which investigates the regulation of Dgat gene by fatty acids in physiologically relevant dose, and in low dose, may provide insight to this finding.

The siRNA-mediated knockdown of *Dgat2* in C2C12 skeletal myotubes decreased glucose uptake into cells, which coincided with impaired Akt signaling and *Glut4* mRNA downregulation in this study. Compared to previous findings, these results regarding glucose uptake and Akt phosphorylation under mediation of *Dgat2* seem to be inconclusive. Transgenic mice overexpressing *Dgat2* in skeletal muscle have shown increased activity of molecules counteracting insulin signaling, such as protein kinase B and protein kinase C lambda, suggesting that these changes are related to increased TAG, ceramides, and unsaturated fatty acids [29]. Another study that utilized an antisense oligonucleotide against *Dgat2* also showed that knocking down *Dgat2* protected against fat-induced hepatic insulin resistance due to decreased DAG amount and protein kinase C epsilon activation [7]. Though this study showed the changes in Akt phosphorylation, the activation of Akt begins with the binding of insulin receptor substrate (IRS) proteins to regulatory subunit of phosphatidylinositol 3 kinase (PI3K) [41,42]. PI3K converts phosphatidylinositol 4,5-biphosphate to phosphatidylinositol (3,4,5)-triphosphate (PIP3), which leads to phosphorylation of PIP3-dependent kinases and finally activates Akt. Phosphatase and tensin homolog (PTEN), which inhibits PI3K, has a role in this process because PTEN dysregulation has been implicated in the regulation of insulin signaling and glucose homeostasis [41]. Another previous study suggested that PTEN has a negative role in TAG accumulation through its inhibition of downstream insulin signaling process and gene expression [43]. Hence, PTEN may have a mutual interplay with the conditions of *Dgat2* knockdown in fatty acid metabolism as well as glucose metabolism. Because this study solely presents data on Akt phosphorylation during the insulin signaling process, we cannot establish the effect of *Dgat2* suppression on upstream insulin signaling preceding Akt phosphorylation. Nevertheless, it seems that the degree of Akt phosphorylation is consistently parallel to the degree of phosphorylation of upstream insulin signaling molecules (IRS, PI3K) as demonstrated in many previous studies [44, 45], and that reduced Akt activity leads to decreased glucose uptake or utilization [42, 45].

A recent study of human skeletal primary myotubes [28] showed no effect on glucose oxidation or glucose uptake by *Dgat2* inhibitor, although it reported decreased glycogen levels to a small extent, suggesting a possible link between *Dgat2*-induced signaling changes and glucose metabolism in skeletal muscle. Hence, the findings of this study need to be confirmed by animal studies and additional experiments that may provide detailed information on glucose metabolism, such as glycogen synthesis and glucose oxidation. In a previous study, the inhibition of *Dgat2* through a chemical inhibitor or siRNA decreased glucose incorporation into TAG as well as mitochondrial oxidation in primary brown adipocytes and immortalized cell lines derived from brown adipose tissues [46], suggesting limited glucose utilization under *Dgat2* deficiency. Because brown adipocytes are high-energy-producing cells that utilize glucose and fatty acids as main substrates [47] and may possess a metabolic pattern similar to skeletal muscle, the results of this study are in line with the previously reported role of *Dgat2* in brown adipocytes. Nevertheless, these results indicate the contribution of *Dgat2* in insulin-derived glucose uptake, and possibly utilization in the skeletal muscle. The results also align with previous findings demonstrating an interplay among intracellular TAG or fatty acid re-esterification and glucose uptake [9, 25].

In this study, the pulse-chase experiment results showed that fatty acid partitioning into total lipids and TAG was reduced without affecting other lipid species, including PL, DAG, and CE under conditions of *Dgat2* knockdown. Increased cellular free acids seem to increase fatty acid availability for beta-oxidation because the remaining fatty acids cannot be esterified to TAG. The data on initial partitioning of radiolabeled fatty acids in this study are in line with the conserved enzymatic function of *Dgat2* gene in finalizing TAG synthesis reported in [14, 15]. Although these enzymes mutually compensate for TAG synthesis, the decreased amount of [1-^14^C]-TAG under *Dgat2* knockdown in this study may correspond to the level of TAG synthetic capacity, which could not be compensated for by *Dgat1*. In this study, the results from chasing radiolabeled fatty acids showed a pattern different from the initial fatty acid partitioning (pulse) results. This result from radioisotope fatty acid metabolic study supported previous findings of *Dgat2* preference toward endogenous synthesized fatty acids in hepatocytes and brown adipose tissue [46, 48]. These data are also consistent with a previous study on human skeletal muscle showing that human DGAT2 affected the re-esterification of fatty acids both exogenously supplied and through lipolysis into cellular lipids in human myotubes [28]. In addition, a more significant reduction in ^14^C-TAG level compared to that during the pulse period and a high-fold increase in free fatty acid level remained during the chase period under *Dgat2* knockdown in this study, indicating that *Dgat1* might not compensate for the loss of *Dgat2* in the re-synthesis of TAG from fatty acids released through lipid breakdown. In addition to TAG, PL level decrease by *Dgat2* suppression may be, in part, due to negative regulation because the main pathway for TAG synthesis is from the glycerophospholipids [15, 49]. Moreover, the limited capacity to synthesize TAG due to *Dgat2* deficiency might limit the formation of TAG precursors, including DAG and PL.

According to previous study using human myotube [28], DGAT1 was dominantly involved in generating TAG by utilizing fatty acids from exogenous source and fatty acids derived from de novo synthesis or lipolysis, while cells treated with DGAT2 inhibitor have shown more pronounced changes in utilizing fatty acids from de novo synthesis (from glycerol-3-phosphate) to synthesize TAG [28]. At the same time, the results indicated that *Dgat1* could not fully substitute *Dgat2* in TAG synthesis from the glycerolipid pathway, suggesting a specific role of *Dgat2* toward endogenous fatty acids over exogenous fatty acids. Furthermore, the findings of this study are supported by previous research, which showed that DGAT2 proteins are co-localized with glycerol-3-phosphate acyltransferase (GPAT) and stearoyl-CoA desaturase (SCD), the main enzymes involved in de novo lipogenesis [48]. In addition, the extent of change in fatty acid incorporation into TAG and the subsequent increase in free fatty acids under *Dgat2* suppression during the chase period tended to be much higher than that during the pulse period, indicating that *Dgat1* completely compensated for the role of TAG synthesis in exogenous fat. Taken together, these data suggest that *Dgat2* may influence both exogenous and endogenous fatty acids, but the role of this gene is more pronounced toward endogenous fatty acids. This result needs to be further validated with experiments for parallel comparison with *Dgat1* knockdown conditions or by investigating the turnover of isotope-labeled glycerol.

Other than intracellular fatty acid metabolism, the aspect of fatty acid channeling into mitochondrial oxidation pathway was investigated in this study. Despite the increase in cellular free fatty acids, *Dgat2*-deficient cells did not increase the ASM level as much as the cellular free fatty acids did. Hence, the decreased incorporation of [1-^14^C]- oleic acids into ASM appears to be due to increased substrate availability, suggesting that *Dgat2* knockdown conditions detour non-esterified fatty acids, which have not been used for TAG synthesis, toward incomplete oxidation. Moreover, the lack of change in CO_2_ production or oxidative gene expression indicated that *Dgat2* suppression did not affect the systemic compartment of substrate oxidation. This finding contradicts previous reports of impaired insulin signaling in transgenic mice overexpressing *Dgat2*, which showed that increased TAG impairs insulin signaling [29]. The timeframe in which glucose uptake was measured corresponded to the timeframe for chasing ^14^C-labeled fatty acids. The results of decreased glucose uptake and Akt phosphorylation coincided with gradually accumulating free fatty acids in *Dgat2* knockdown cells, indicating the possibility that cellular free fatty acids impair insulin signaling or limit the capacity of cells to uptake glucose through the production of inflammatory mediators. Moreover, *Dgat2* is physically associated with lipid droplets and co-localizes to the mitochondrial membrane [27, 50]; therefore, the finding may further support the role of *Dgat2* in the oxidation of exogenous or endogenous fatty acids.

Although we could not investigate the aspect of *Dgat1* suppression in fatty acid metabolism in this study, the results of previous studies provide a possible comparison point. Skeletal muscle-specific overexpression of *Dgat1* increased Akt phosphorylation and membrane bound *Glut4* expression more than two-folds despite high accumulation of TAG. In addition, *Dgat1* transgenic mice increased intracellular TAG level, but also increased fatty usage for fatty acid oxidation, and these changes were comparable to metabolic changes in exercised mice which have shown increased TAG level with increased substrate usage, which led to lesser accumulation of lipotoxic fatty acid derivatives such as ceramides and DAG [25]. In human skeletal muscle cells, DGAT2 inhibitor reduced TAG synthesis and LD formation from exogenous fatty acids much less compared to DGAT1 inhibitor, which indicates the more important role of DGAT1 toward exogenous fatty acids compared to that of DGAT2. Insulin-induced glycogen synthesis from C14-labeled glucose was only significantly decreased in DGAT2 inhibitor-treated cells [28]. In addition, our data from C2C12 skeletal myotubes are consistent with findings observed in other tissues including hepatocytes and brown adipocytes, which show that TAG synthesis from exogenous fatty acids is primarily mediated by *Dgat1* [48, 51]. DGAT2 inhibition of primary mouse and human hepatocytes has shown more reduction in de novo synthesized fatty acids channeling into triglyceride than in channeling fatty acids into exogenously added oleic acids. However, the inhibition of DGAT1 affects on a similar level the incorporation of preformed oleic acids into TAG [51]. A similar study also implied that DGAT1 seems to channel fatty acids preferably toward mitochondrial oxidation more than DGAT2 does in HepG2 cells [48]. Hence, the mechanism of mediating impaired insulin signaling and glucose uptake coincident with TAG under *Dgat2* suppression would be different from the mechanism of how *Dgat1* mediates insulin signaling and glucose metabolism.

Because we only measured the metabolism of fatty acids from exogenous sources, or from lipolysis, we cannot reach a conclusion on a muscle-specific role of *Dgat2*, which was previously reported in the context of its preference toward endogenous fatty acids. To bolster evidence of the inclination of *Dgat2* to metabolize endogenous fatty acids, the aspects of metabolic changes of fatty acids from glycerol-3-phosphate or -acetate under *Dgat2* deficiency or augmentation need to be investigated. Although similar or overlapping *Dgat2* function was reported among tissues, the consequences of fatty acid metabolism changes could vary according to the function of each tissue. Hence, the consequences of *Dgat2* and *Dgat1* action in muscle energy metabolism and expansion to muscle contraction and energy production need to be further investigated in depth. Based on the glucose uptake results and intracellular fatty acid channeling in this study, *Dgat2* suppression may affect glucose uptake, partly by converting free fatty acids to other lipid metabolites that affect insulin signaling. Further lipidomic analysis of fatty acid metabolites under *Dgat2* suppressed condition may provide some insights into this issue.

Based on the similar role of DGAT genes in TAG accumulation, clinical application of inhibitors targeting these two DGAT isoforms has been attempted in nonalcoholic fatty liver disease. DGAT1 inhibition seems to have an undesirable side effect on the gastrointestinal tract, which limits further clinical development of DGAT1 inhibitor [52]. So far, DGAT2 inhibition has not been attempted in a clinical setting but was reported as a well-tolerated therapeutic option for patients with nonalcoholic fatty liver disease [53]. In addition, DGAT2 mutations are associated with the phenotype of impaired muscle structure and function (*e.g.*, Charcot-Marie-Tooth disease) in humans [54] and cellular transformation properties in several types of human cancer [55]. The findings of this study will add more insight in the application of DGAT inhibitor in risk prediction of several metabolic diseases and in phenotype identification according to their genotype match.

In summary, we investigated the effect of transient *Dgat2* knockdown in glucose uptake and fatty acid channeling in C2C12 skeletal muscle cells. The results suggest that *Dgat2* metabolizes exogenously and endogenously generated fatty acids. Because *Dgat2* may act differently depending on the muscle fiber types or the status of the muscle fiber (resting vs. exercising) [56], the role of *Dgat2* in skeletal myotubes under different energetic conditions and compared with *Dgat1* needs to be further elucidated. Taken together with the different results in the degree of fatty acid incorporation into TAG between the pulse and chase periods, and the higher stimulation of *Dgat2* expression in response to palmitic acid over oleic acid, *Dgat2* seems to have a greater preference toward endogenous fatty acids over exogenous fatty acids in skeletal muscle, which is in line with previously reported results in other cell types. Although a parallel comparison needs to be performed under the *Dgat1* conditions, these data suggest a unique function of *Dgat2* in fatty acid and TAG metabolism in C2C12 cells.

## Figures and Tables

**Table 1:** List of primer sequences for qPCR.

Gene	Forward primer (5’-3’)	Reverse primer (5’-3’)
*Acsl1*	ACCATGTACGATGGCTTCCA	TCATAGGGCTGGTTTGGCTT
*Acsl6*	ACGAGGACAGGACAAAGGAG	CTCTGG CGCAACATATTCCC
*Glut4*	TTGGGAAGGAAAAGGGCTAT	GAGGAACCGTCCAAGAATGA
*Dgat1*	TTCCGCCTCTGGGCATT	AGAATCGGCCCACAATCCA
*Dgat2*	GCT GGC ATT TGA CTG GAA CA	AGT AGT CTC GGA AGT AGC GC
*Ppar-α*	ATGTGTCGCCTTCTTGCTCT	ATCTACTGCCTGGGGACCTT
*Cpt1-α*	GATGTGGACCTGCATTCCTT	TCCTTGTAATGTGCGAGCTG
*Ucp2*	GCACTGTCGAAGCCTACAAG	TCATAGGTCACCAGCTCAGC
*Rpl32*	AACCCAGAGGCATTGACAAC	ATTGTGGACCAGGAACTTGC
